# Trabecular bone score and sclerostin concentrations in patients with primary adrenal insufficiency

**DOI:** 10.3389/fendo.2022.996157

**Published:** 2022-11-02

**Authors:** Aleksandra Zdrojowy-Wełna, Jowita Halupczok-Żyła, Natalia Słoka, Joanna Syrycka, Łukasz Gojny, Marek Bolanowski

**Affiliations:** ^1^ Department of Endocrinology, Diabetes and Isotope Therapy, Wroclaw Medical University, Wroclaw, Poland; ^2^ Laboratory of Molecular Endocrinology, Department of Endocrinology, Diabetes and Isotope Therapy, Wroclaw Medical University, Wroclaw, Poland

**Keywords:** primary adrenal insufficiency, sclerostin, trabecular bone score, bone mineral density, Addison’s disease

## Abstract

**Background:**

Patients with primary adrenal insufficiency need lifelong replacement therapy with glucocorticoids and mineralocorticoids, which may influence their bone quality.

**Aim:**

The aim of the study was to evaluate densitometry parameters, trabecular bone score and sclerostin concentrations in patients with primary adrenal insufficiency in comparison to control group.

**Materials and methods:**

We included 29 patients (62% females) with diagnose of autoimmune primary adrenal insufficiency (mean age 49.7 ± 11.7 years, mean duration of the disease 13.2± 13.6 years) and 33 healthy subjects (adjusted with age, sex and body mass index). Bone mineral density at the femoral neck, lumbar spine, total body and trabecular bone score were evaluated. Serum sclerostin concentrations were measured.

**Results:**

There were no significant differences in densitometry parameters (T-score, Z-score, bone mineral density in all locations) as well as in trabecular bone score in patients with adrenal insufficiency in comparison to control group. Mean serum sclerostin concentration was significantly higher in patients with adrenal insufficiency than in control group (44.7 ± 23.5 vs 30.7 ± 10.4 pmol/l, p=0.006). There was a negative correlation between trabecular bone score and the duration of adrenal insufficiency and age, also a negative correlation between femoral neck and total densitometry parameters and 24-hour urine cortisol as a marker of hydrocortisone daily dose in patients with adrenal insufficiency.

**Conclusions:**

The bone status in patients with primary adrenal insufficiency was not impaired in comparison to control group, while sclerostin concentration was higher. The duration of the disease and higher hydrocortisone doses may affect negatively bone status.

## Introduction

Primary adrenal insufficiency (Addison’s disease) is a rare disorder, characterized by an inability of the adrenal cortex to produce sufficient amounts of glucocorticoids and mineralocorticoids. The prevalence in European countries is about 100-140 cases per million (1, 2) and it is increasing (3). Despite receiving hormonal replacement therapy, patients suffer from impaired quality of life (4–6) and a higher mortality rate - especially if diagnosed at a young age (7–9). It may be a consequence of coexisting autoimmune disorders in about 60% of patients (10), but also adrenal crises (8), malignancies, infections, and cardiovascular complications (7, 11). Another important problem is the risk of osteoporosis, but data concerning patients with primary adrenal insufficiency are inconsistent or even contradictory. The cross-sectional study comprising 292 patients from Norway, Britain, and New Zealand showed a reduced bone mineral density (BMD) at the femoral neck and lumbar spine (12). Another population-based cohort study from Sweden showed an increased risk of hip fracture in patients in comparison to control group, especially females diagnosed with autoimmune primary adrenal insufficiency < 50 years of age (13). There are also studies reporting no significant impairment of BMD (14, 15).

It has been shown, that standard glucocorticoid replacement therapy provides doses higher than produced in healthy adrenal glands, which may affect bone status (16). Hypercortisolemia leads to increased fracture risk due to inhibition of calcium absorption, impaired bone formation, and increased resorption, especially in bone with high trabecular content (17). One of the mediators of the adverse effect of glucocorticoids on bone may be the influence on Wnt/βcatenin signaling, a pathway known to promote differentiation of mesenchymal stem cells into osteoblasts. Canonical Wnt signaling also stimulates osteoblast maturation and survival of osteoblasts and osteocytes, while decreasing osteoclast generation (18). Glucocorticoids inhibited intracellular Wnt signaling, resulting in the suppression of osteoblast differentiation *in vitro* (19, 20). Sclerostin, encoded by the Sost gene, is a protein secreted mainly by osteocytes, that inhibits Wnt/βcatenin signaling mainly through interaction with the lipoprotein receptor-related protein (LRP) family (18). In mice, glucocorticoids increased the expression of Sost gene, while knockout mice lacking Sost were protected from bone loss in conditions of glucocorticoid excess (21). Additionally, treatment with antibodies against sclerostin in mice prevented glucocorticoid-induced osteoporosis (22). In line with animal results, in a study comprising patients treated with more than 7.5 mg of prednisolone per day for a year, sclerostin concentration increased significantly and correlated with glucocorticoid dose (23). On the other hand, in 21 patients with chronic endogenous Cushing syndrome, sclerostin concentration in serum was significantly reduced in comparison to control group, and increased after treatment (24). In a recent randomized intervention study, 64 healthy males were given a placebo or prednisolone 7.5 mg daily, or prednisolone 30 mg daily for 2 weeks. Compared with placebo, prednisolone high-dose decreased serum sclerostin concentrations, while low-dose did not alter sclerostin concentrations (25). Another study did not show any significant influence of short-term hypercortisolemia after adrenocorticotropin (ACTH) infusion on sclerostin concentrations in 17 healthy subjects (26). In summary, studies *in vitro* and in mice suggest that inhibiting the canonical Wnt/β-catenin signaling pathway, especially through increased expression of its antagonists like sclerostin, is an important factor contributing to glucocorticoid-induced osteoporosis. In humans results are inconsistent. The duration and type of used glucocorticoid may be crucial. Some authors also suggest, that chronic hypercortisolemia affects the number and function of osteocytes, cells that are the main source of sclerostin, rather than affects directly sclerostin concentration (24).

The trabecular bone score (TBS) is a new diagnostic method, providing indirect information on trabecular bone microarchitecture (27). It is obtained from the computed evaluation of pixel grey-level variations in lumbar spine dual-energy X-ray absorptiometry (DXA) images. TBS is correlated with trabecular bone volume, the number of trabeculae and their connectivity (28). It has been shown, that in patients with endogenous hypercortisolemia TBS was significantly lower than in control group and correlated with disease duration (29).

To our best knowledge, there was no study evaluating TBS and sclerostin concentrations in patients with primary adrenal insufficiency so far. It may be important because of glucocorticoid deficiency – a mainstay of the disease, but also common overtreatment with hydrocortisone in the replacement therapy. There are other factors contributing to osteoporosis in patients with primary adrenal insufficiency, like deficiency of adrenal androgens (30) or other autoimmune diseases that need to be taken into consideration.

In this context, the aim of our study was to evaluate TBS and sclerostin serum concentrations in patients with autoimmune primary adrenal insufficiency.

## Materials and methods

### Subjects

We included 29 patients with diagnose of autoimmune primary adrenal insufficiency (18 women, 11 men; mean age 49.7 ± 11.7 years) and sex-, age-, body mass index (BMI) matched 33 controls (20 women, 13 men, mean age 54.8 ± 9.5 years), who were patients of Department of Endocrinology, Diabetes and Isotope Therapy in Wrocław, Poland, between February 2020 and February 2022. All subsequent patients followed up in our department were included in the study. Only one patient denied to take part in the study. Three patients were newly diagnosed with Addison`s disease, so we waited 6 months of stable therapy to perform examinations. The patients with longer duration of the disease had stable doses of replacement therapy for at least a year. In case of infections doses were increased for a short period of time, however 3 months before our examinations doses were not modified.

According to the Endocrine Society Clinical Guidelines, the primary adrenal insufficiency diagnosis was established on the basis of the following criteria: peak cortisol concentrations below 18 µg/dl at 30 or 60 minutes after corticotropin stimulation test (250 µg) as a gold standard, alternatively morning cortisol concentration below 5 µg/dl in combination with ACTH concentration increased twofold the upper reference range as a preliminary test (31). The inclusion criteria was the diagnosis of autoimmune primary adrenal insufficiency treated with a hormonal replacement therapy for at least 3 months. The autoimmune cause of the adrenal insufficiency was made on the basis of positive testing for autoantibodies against adrenal 21 hydroxylase or coexistence of other autoimmune diseases, without any other known cause of adrenal insufficiency.

A thorough medical history was taken, including detailed information about hormonal replacement therapy (current doses and mean doses for the last 3 months) and osteoporotic risk factors. The physical examination included measurement of height in cm, weight in kg, on this basis BMI was calculated. The patients did not report changes in body height. The waist circumference was measured with tape, halfway between the lowest rib and the top of the hipbone. The hip circumference was measured with tape, at the widest part of the buttocks.

We collected concentrations of following parameters in blood: morning ACTH, direct renin concentration, 25(OH) vitamin D, dehydroepiandrosterone sulfate (DHEA-S), estradiol (E2), follicle-stimulating hormone (FSH), luteinizing hormone (LH), total testosterone (T), sex hormone binding globulin (SHBG), thyroid-stimulating hormone (TSH), free thyroxine (fT4), insulin-like growth factor (IGF-1), parathormone (PTH), calcium, and alkaline phosphatase (ALP). The free androgen index (FAI) was calculated as the ratio of T divided by SHBG and multiplied by 100. We also measured urinary free cortisol (UFC). An oral glucose tolerance test (OGTT) with 75 g glucose in patients without known diabetes was conducted.

In the group with adrenal insufficiency 5 patients had type 1 diabetes treated with insulin, one patient had type 2 diabetes, 21 patients had primary autoimmune hypothyroidism treated with L-thyroxine. In the control group, 4 patients had type 2 diabetes, 4 patients had autoimmune hypothyroidism treated with L-thyroxine. The doses of L-thyroxine and insulin were stable for at least 3 months, there were no exacerbations of concomitant diseases for at least 3 months.

In patient`s group there were 10 postmenopausal women (56%), four of them received hormonal replacement therapy, 8 women were premenopausal (44%). In the control group there were 12 postmenopausal women (60%), none of them received hormonal replacement therapy, 8 women were premenopausal (40%).

On the basis of the WHO densitometric criteria (32), in the adrenal insufficiency group at the lumbar spine 13 patients had osteopenia, 3 had osteoporosis. At the femoral neck 14 patients had osteopenia, 2 had osteoporosis. In the control group at the lumbar spine 8 subjects had osteopenia, 3 had osteoporosis. At the femoral neck 14 controls had osteopenia, no one had osteoporosis.

No patient from the adrenal insufficiency group or control group had pituitary insufficiency, hyperprolactinemia, hyperparathyroidism (based on current hormonal measurements) or osteoporotic fracture (based on medical history and clinical examination). All patients had normal kidney and liver function.

### Laboratory examinations

The ACTH, LH, FSH, E2, T, DHEA-S, PTH,fT4 concentrations were measured by chemiluminescence immunoassay method using Immulite 2000 (Siemens Healthcare Diagnostics, USA). Following reference ranges were used: ACTH <46 pg/ml (analytical sensitivity: 5.0 pg/ml); LH premenopausal women: follicular phase 1.1-11.6 mIU/ml, ovulation phase 17.0-77.0 mIU/ml, luteal phase 0.7-14.7 mIU/ml; postmenopausal women: 11.3-39.8 mIU/ml; men: 0.8-7.6 mIU/ml (analytical sensitivity: 0.05 mIU/ml); FSH premenopausal women follicular phase 2.8-11.3 mIU/ml, ovulation phase 5.8-21.0 mIU/ml, luteal phase 1.2-9.0 mIU/ml; postmenopausal women 21.3-153.0 mIU/ml; men 0.7-11.1 mIU/ml (analytical sensitivity: 0.1 mIU/ml); E2 premenopausal women: follicular phase <160.0 pg/ml, ovulation phase 34–400.0 pg/ml, luteal phase 27.0–246.0 pg/ml; postmenopausal women <30.0 pg/ml; men <56.0 pg/ml (analytical sensitivity: 15.0 pg/ml); T premenopausal women 0.2–0.72 ng/ml, postmenopausal women 0.2–0.43 ng/ml, men 0.72–8.53 ng/ml, >50 years 1.29–7.67 ng/ml (analytical sensitivity: 0.15 ng/ml); DHEA-S: women 35.0–430.0 µg/dl; men 80.0–560.0 µg/dl (analytical sensitivity: 3.0 µg/dl), PTH 11.0-76.0 pg/ml (analytical sensitivity: 3.0 pg/ml), fT4 10-22 pmol/l (analytical sensitivity: 0.5 pmol/l).

UFC was measured using a radioimmunoassay method (Immunotech, Beckman Coulter Inc., Prague, Czech Republic), reference range in control group 14.0–120.0 μg/24 h. There is no available reference range in patients receiving hydrocortisone replacement therapy.

25(OH)D concentrations were measured by chemiluminescent immunoassay using Architect i1000 (Abbott Laboratories, USA), reference ranges: vitamin D deficiency <20 ng/ml, suboptimal status 20–30 ng/ml, optimal status 30–70 ng/ml. Limit of detection (LOD) was 2.2 ng/ml, limit of quantitation (LOQ) was 2.4 ng/ml.

Serum calcium and alkaline phosphatase were measured using colorimetric assays on an Architect c4000 (Abbott Laboratories, USA). Reference ranges were as follows: calcium 8.4–10.5 mg/dL (LOD: 0.5 mg/dl; LOQ: 1.0 mg/dl); alkaline phosphatase 40–150 IU/l (LOD: 5.0 IU/l; LOQ: 5.0 IU/l).

Sclerostin in the serum was measured using a sandwich ELISA kit from Biomedica (Biomedica Wien, Austria). The analysis was performed in accordance with the attached protocol in sample duplicate during a single session.

### BMD and TBS assessment

BMD at the femoral neck, lumbar spine (L1-L4), and total body were evaluated using the DXA technique (Hologic Horizon A densitometer). The coefficients of variation (CV) were as follows: females - femoral neck 1.69%, lumbar spine 1,6%, total body 1%; males - femoral neck 1.8%, lumbar spine 1.3%, total body 1%. Results were presented as BMD (g/cm^2^), T-score, and Z-score. According to WHO criteria in postmenopausal women and men aged >50 years, we used the following categories: T-score ≥-1 standard deviations (SD) – normal; T-score between -1 and -2.5 SD – osteopenia; T-score ≤-2.5 SD – osteoporosis. In premenopausal women or men aged < 50 years we used Z-score: values of -2.0 SD or lower are stated “below the expected range for age” and those above -2.0 SD “within the expected range for age (32).

TBS values were obtained from lumbar spine DXA images using TBS iNsight software, version 3.0.3.0 (Med-Imaps, Pessac, France). In line with other studies, the following criteria were used: TBS ≥1.31 normal, 1.31–1.23 partially degraded microarchitecture, ≤1.23 degraded microarchitecture (33).

### Statistical analysis

Statistical analysis was performed using Statistica software for Windows, version 13.3 (StatSoft),. The mean, median, standard deviation (SD), and interquartile ranges (IQR) were determined for all variables. The Shapiro–Wilk test was used to check the normality of the data distribution. Student’s t-test or Mann-Whitney test was applied to compare quantitative variables, whereas categorical variables were compared by the chi-square test or Fisher’s exact test. Correlations between parameters were evaluated using Pearson’s test or Spearman’s rank correlation test as appropriate. Moreover, a multiple regression analysis was used to identify the predictors of TBS. P-value <0.05 was considered statistically significant

### Ethics

The Bioethics Committee of Wroclaw Medical University approved the protocol of the study. All subjects signed informed consent forms in accordance with the Declaration of Helsinki. The participants provided their written informed consent to participate in this study.

## Results

The general characteristics of the group with primary adrenal insufficiency and controls are presented in **Table 1**. The adrenal insufficiency group comprised 29 patients (62% females, mean age 49.7 ± 11.7 years) and the control group comprised 33 subjects (61% women, mean age 54.8 ± 9.5 years). There were no significant differences in age, sex, body mass, and BMI between the studied groups. The patient`s group and controls did not differ significantly in case of the frequency of postmenopausal women.

**Table 1:** Characteristics of the patients with primary adrenal insufficiency and controls.

	Adrenal insufficiency (n = 29)			Control group (n = 33)			p-value
	Mean ± SD	Median	IQR	Mean ± SD	Median	IQR	
Age (years)	49.7 ± 11.7	48	39-58	54.8 ± 9.5	55	46-64	0.052
Body mass (kg)	72.8 ± 16.3	70	59-82	78.7 ± 11.4	80	70-85	0.098
BMI (kg/m^2^)	25.6 ± 4.4	26.4	21.9-28.7	27.4 ± 2.7	27.5	25-29.2	0.064
ACTH (pg/ml)	584.5 ± 477	421	152-1184	19.4 ± 19.2	14.7	10.6-20.1	**<0.000**
UFC (µg/day)	88.5 ± 61.6	72.8	39.9-123.9	49.7 ± 23.3	43.8	34.3-59.6	**0.013**
DHEA-S (µmol/l)	24.1 ± 17.7	15	15-28.6	124.04 ± 67.5	112	77.2-158.0	**<0.000**
25(OH) vitamin D (ng/ml)	31.3 ± 14.0	28.8	22.2-40.5	25.9 ± 6.5	25.6	22.1-29.5	**0.049**
PTH (pg/ml)	28.7 ± 12.8	26.2	19.0-37.3	41.8 ± 21.7	34.9	25-57	**0.019**
ALP (U/l)	55.1 ± 15.1	52.5	43.4 ± 65.0	63.2 ± 17.7	61	50-72	0.059
SCL (pmol/l)	44.7 ± 23.5	35.4	30.5-48.9	30.7 ± 10.4	28.7	24.3-35.5	**0.006**
LS T-score	-0.83 ± 1.3	-0.9	-1.9-0.4	-0.90 ± 1.0	-0.5	-1.5-(-)0.1	0.817
LS Z-score	-0.18 ± 1.3	-0.2	-1.1-0.8	0.06 ± 0.9	0.1	-0.6-0.6	0.371
LS BMD (g/cm^2^)	0.97 ± 0.2	1.0	0.9-1.1	0.96 ± 0.1	1.0	0.9-1.0	0.744
FN T-score	-0.90 ± 0.9	-1.1	-1.5-(-)0.5	-0.78 ± 0.9	-0.8	-1.5-(-)0.2	0.617
FN Z-score	-0.17 ± 0.9	-0.2	-0.8-0.3	0.13 ± 0.9	0.2	-0.5-0.5	0.179
FN BMD (g/cm^2^)	-0.77 ± 0.1	0.8	0.7-0.9	0.80 ± 0.1	0.8	0.7-0.9	0.380
TBS	1.32 ± 0.1	1.3	1.25-1.37	1.30 ± 0.1	1.29	1.22-1.39	0.630

SD, standard deviations; IQR, interquartile range; BMI, body mass index; ALP, alkaline phosphatase; SCL, sclerostin; BMD, bone mineral density; LS, lumbar spine; FN, femoral neck; TBS, trabecular bone score.

Bold values are considered statistically significant (p < 0.05).

Mean plasma ACTH concentration and UFC were significantly higher in group with the adrenal insufficiency (p<0.000 and p=0.013, respectively), while controls had higher DHEA-S concentration (p<0.000).

The characteristic of disease duration and hormonal replacement therapy in the adrenal insufficiency group is presented in **Table 2**. Mean disease duration was 13.2 ± 13.6 years, mean age at diagnosis was 36.6 ± 10.9 years. All patients were treated with hydrocortisone (mean daily dose 25.8 ± 6.2 mg), one patient received also 0.125 mg of dexamethasone per day. 19 patients received ≥ 25 mg of hydrocortisone daily. 26 patients received fludrocortisone (mean dose 0.07 ± 0.06 mg).

**Table 2:** Characteristics of the patients with autoimmune primary adrenal insufficiency (n = 29).

	Mean ± SD	Median	IQR
Duration of the disease (years)	13.2 ± 13.6	6	3-26
Age at diagnosis	36.6 ± 10.9	36	29-41
Daily dose of hydrocortisone (mg)	25.8 ± 6.2	25	20-30
Mean daily dose of hydrocortisone from last 6 months (mg)	26.2 ± 6.1	28	22.5-30
Daily dose of fludrocortisone (mg)	0.07 ± 0.06	0.05	0.025-0.1
Mean daily dose of fludrocortisone from last 6 months (mg)	0.07 ± 0.06	0.05	0.034-0.1
Sodium (mmol/l)	139.4 ± 3.2	139	137-141
Potassium (mmol/l)	4.2 ± 0.3	4.2	4.0-4.5
Fasting glucose (mg/dl)	93.3 ± 23	88	81-93
Systolic blood pressure (mmHg)	128 ± 14.4	125	120-140
Diastolic blood pressure (mmHg)	79 ± 10.4	80	75-85

SD, standard deviations; IQR, interquartile range.

**Figure 1:**
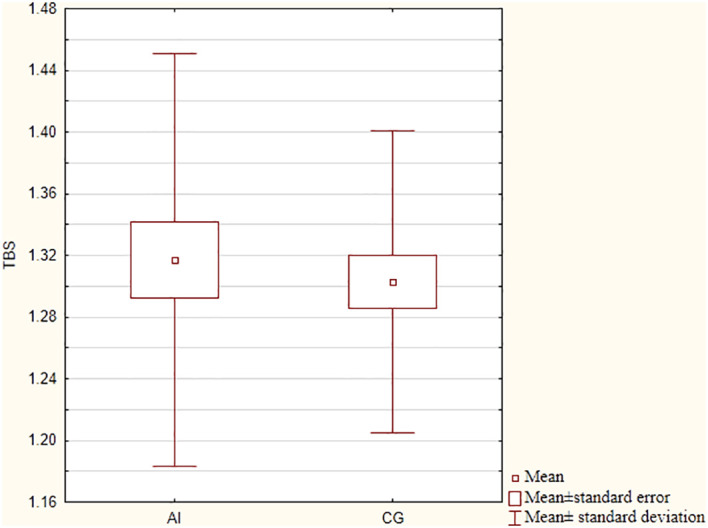
TBS values in patients with primary adrenal insufficiency and control group. TBS, trabecular bone score; AI, adrenal insufficiency group; CG, control group.

There were no significant differences in BMD, T-score or Z-score values at the lumbar spine and femoral neck between the studied groups. TBS also did not vary between groups (p=0.63), as presented in **Figure 1**. Mean TBS values in both groups indicated partially degraded bone microarchitecture (between 1.31 and 1.23).

**Figure 2:**
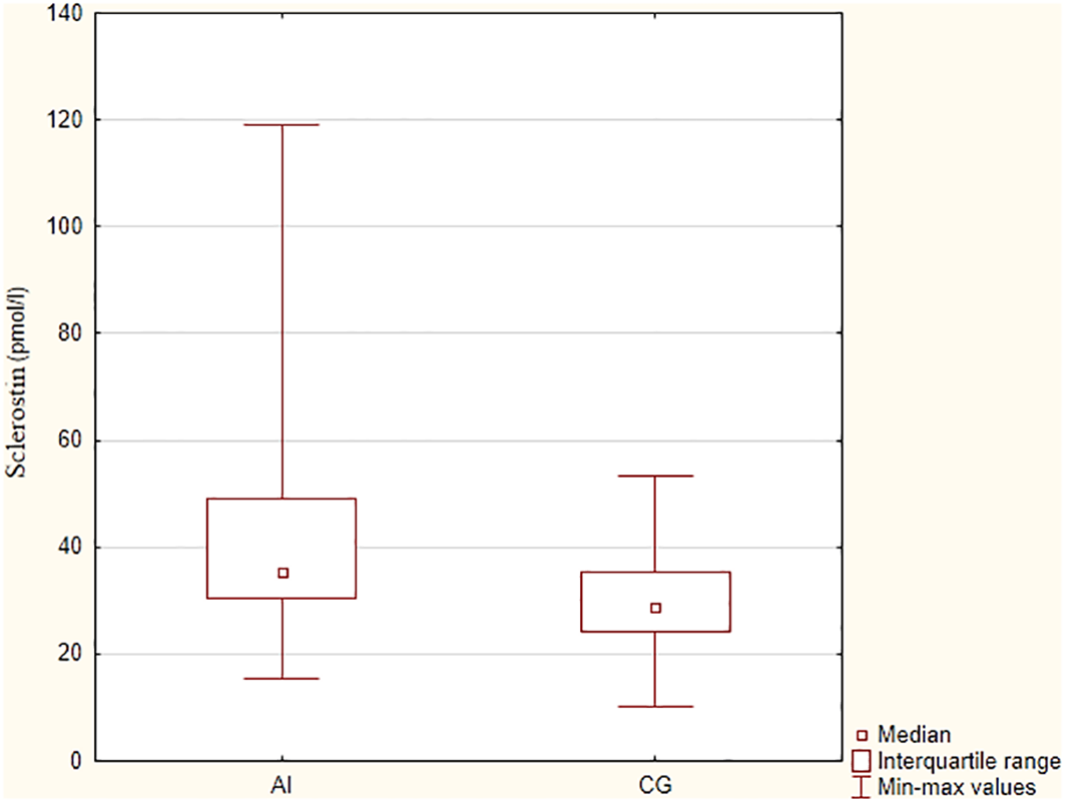
Sclerostin concentrations in patients with primary adrenal insufficiency and control group. AI – adrenal insufficiency group, CG – control group.

The sclerostin concentration was significantly higher in the group with adrenal insufficiency in comparison to the control group (mean values 44.7 ± 23.5 pmol/l vs 30.7 ± 10.4 pmol/l, p=0.006), as presented in **Figure 2**. There were no significant differences in sclerostin concentration, TBS, BMD at the LS and FN between patients receiving higher vs lower doses of hydrocortisone (we divided groups according to mean daily dose 25.8 mg and median of the daily dose – 25 mg, p> 0.05).

We performed further analyses to assess correlations between sclerostin and other clinical parameters. We only found correlation with serum fT4 concentration (R=0.486, p=0.009; no significant correlation with serum TSH). Other clinical factors (hydrocortisone dose, duration of adrenal insufficiency, duration of autoimmune hypothyroidism) and hormones were not associated significantly with sclerostin serum concentration.

There were no significant differences in calcium or alkaline phosphatase concentrations between patients and controls, but vitamin D concentration was higher in the adrenal insufficiency group (p=0.049), while controls had higher PTH concentrations (p=0.019).

In patients with primary adrenal insufficiency further analyses were conducted to assess correlations between TBS/densitometry parameters with other clinical characteristics (**Table 3**). TBS correlated negatively with age (p=0.004), the duration of the disease (p=0.009) and gonadotropin concentrations (p=0.001 for FSH, p=0.038 for LH). There was a positive correlation between TBS and BMD at the lumbar spine (p=0.000) and total body (p=0.011). Further, we conducted a multiple regression analysis to determine the influence of clinical factors and hormones on TBS value. At the beginning, we included factors such as age, disease duration, DHEA-S, FSH, LH, TSH, PTH, IGF-1, BMI. The final model comprised age and LH concentration. The multiple regression analysis revealed that age (ß - (–)0.0042, standard error (SE)- 0.00197, p=0.042) and LH concentration (ß - (-)0.0035, SE - 0.00172, p=0.049) are independent predictors of TBS.

**Table 3:** Correlations between TBS/densitometry parameters/sclerostin and other clinical characteristics in 29 patients with autoimmune primary adrenal insufficiency.

		R	p
TBS			
	age	-0.523	0.004
	disease duration	-0.479	0.009
	LS BMD	0.615	0.000
	total body BMD	0.475	0.011
	FSH	-0.572	0.001
	LH	-0.387	0.038
LS BMD			
	DHEA-S	0.372	0.046
	FSH	-0.628	0.0003
FN T score			
	UFC	-0.654	0.0003
	FSH	-0.519	0.004
	estradiol	0.381	0.045
	FAI	0.482	0.013
FN Z score			
	UFC	-0.448	0.022
	FAI	0.504	0.009
FN BMD			
	UFC	-0.664	0.0002
	DHEA-S	0.622	0.0003
	FSH	-0.595	0.001
	testosterone	0.515	0.004
	FAI	0.620	0.001
total body T score			
	UFC	-0.476	0.016
	estradiol	0.384	0.048
total body BMD			
	UFC	-0.462	0.020
	DHEA-S	0.603	0.0007
	FSH	-0.733	0.000009
	testosterone	0.529	0.004
sclerostin			
	fT4	0.486	0.009

fT4, free thyroxine concentration; BMD, bone mineral density; DHEA-S, dehydroepiandrosterone-sulfate; FAI, free androgen index; FSH, foliculotropin; LH, lutropin; TBS, trabecular bone score; TBS, trabecular bone score; UFC, urinary free cortisol.

BMD in all examined locations (LS, FN, total body) correlated positively with serum DHEA-S concentrations and negatively with gonadotropin concentrations (**Table 3**, data not shown for LH). Gonadotropin concentrations were also negatively associated with T-score and Z-score in total body densitometry (T-score and FSH: p=0.00003, R=(-)0.702). Estradiol concentrations were positively correlated with T-score value at the femoral neck (p=0.045) and total body (p=0.048).

DHEA-S was also positively associated with T-score and Z-score at the femoral neck (p=0.016 and p=0.02, respectively) and total body T-score (p=0.016). Testosterone correlated positively with BMD in FN and total body. FAI was positively associated with FN T-score, Z-score, and BMD.

UFC was significantly negatively associated with densitometry parameters in FN (T-score, Z-score, BMD) and total body (BMD, T-score) (**Table 3**).

Additionally, we analyzed the influence of most prevalent concomitant autoimmune diseases (autoimmune hypothyroidism in 21 patients and autoimmune diabetes in 5 patients with primary adrenal insufficiency). There were no significant differences in sclerostin concentration, TBS and BMD between patients with and without diabetes in subject with primary adrenal insufficiency (p>0.05). Similarly, we did not observe significant differences in sclerostin, TBS, BMD between patients with and without the diagnose of autoimmune hypothyroidism. There was also no correlation between the duration of autoimmune hypothyroidism and sclerostin (R=0.186, p= 0.34), TBS (R=-0.046, p=0.81), BMD, T-score and Z-score.

## Discussion

We found no significant difference between TBS or BMD in patients with autoimmune primary adrenal insufficiency in comparison to controls. To our best knowledge, this is the first report of TBS in patients with Addison’s disease. Previous studies examined BMD or osteoporotic fractures in this group of patients with varying results. The biggest cross-sectional study from Norway, UK and New Zealand showed reduced BMD at the FN and LS in comparison to the control group (12). Some authors reported no significant difference in BMD between patients with Addison's disease and healthy subjects, however, numbers of patients were mostly small (14, 15). Some studies showed also an increased prevalence of vertebral and hip fractures in patients with primary adrenal insufficiency (13, 34). Maybe our study group was too small to demonstrate differences in TBS/BMD values between patients and controls because even in larger cohorts those differences were subtle (12). Another interesting aspect is that in our study vitamin D concentration was higher in the adrenal insufficiency group, while controls had higher PTH concentrations. It may suggest, that patients with Addison`s disease used more effective vitamin D supplementation than controls.

The evaluation of osteoporosis risk in patients with primary adrenal insufficiency is difficult because there are many contributing factors. The first of them is overtreatment with glucocorticoids. Studies with the use of thermospray liquid chromatography-mass spectrometry showed that daily steroid production by healthy adrenal glands is about 9.9 ± 2.7 mg/day, (5.7 mg/m^2^/day) (16) and the suggested dose in replacement therapy is 15-20 mg of hydrocortisone per day by many authors (35). According to European Adrenal Insufficiency Registry (EU-AIR) data, 42% of patients with adrenal insufficiency received daily 20 to 25 mg of hydrocortisone, while 12.6% over 30 mg per day (36). In our group mean daily dose of hydrocortisone was 26.2 ± 6.1 mg, while 19 patients received 25 mg or more. We found that UFC was significantly higher in patients receiving hydrocortisone than in controls. Espiard et al. have shown, that cortisol and all of its metabolites correlated positively with daily hydrocortisone dose in patients with Addison`s disease and UFC was 3-fold higher than in controls (37) UFC in patients with Addison`s disease may be used as a marker of glucocorticoid dose. We found a negative correlation between UFC and densitometry parameters at the FN and total body. Similarly, in the Norwegian Registry of Addison’s disease the Z-scores at the FN, total hip, and total body, but not those at the lumbar spine, were significantly associated with weight-adjusted glucocorticoid dose (12) We found no association between TBS and glucocorticoid dose or UFC. It is known, that hypercortisolemia has a negative impact on bone by many mechanisms, not only reduction in mineralization (17). That is why sole BMD evaluation is not sufficient in estimating glucocorticoid-related fracture risk. TBS has strong positive correlations with the trabecular bone volume to tissue volume ratio, number of trabeculae, and their connectivity and stiffness (38). In this context, TBS appears to be a valuable tool in assessing the bone status and estimating fracture risk in patients with glucocorticoid-induced osteoporosis. It has been shown that in patients with endogenous hypercortisolemia TBS values were significantly lower than in controls, moreover, subjects with Cushing`s syndrome with fractures had low TBS values (29). However, the effect of exogenous glucocorticoids on bone may be different, because it is modulated by various mechanisms at a tissue level, like variations in the expression and sensitivity of the glucocorticoid receptors (GRs), the action of transmembrane transporters, and enzymatic metabolism of glucocorticoids to more or less active forms by 11β-hydroxysteroid dehydrogenases (11β-HSDs) (17). It has been shown, that a common polymorphism in the efflux transporter P-glycoprotein was associated with reduced BMD and increased susceptibility to glucocorticoid-induced osteoporosis in patients with Addison`s disease (12). It suggests that other factors contributing to glucocorticoid action on tissue level may play an important role in glucocorticoid induced osteoporosis development. In our group more than half of the patients received over 25 mg hydrocortisone daily, suggesting overtreatment, while TBS/BMD values did not differ from the control group. We also did not find differences in bone parameters between groups on higher vs lower doses of hydrocortisone. Maybe factors influencing steroids metabolism played important role in this case. This aspect needs further studies.

In our group, TBS correlated negatively with the duration of the disease. It stays in accordance with the previous studies showing that patients with a longer history of primary adrenal insufficiency had a higher prevalence of vertebral fractures (34). Also, the age of patients and postmenopausal status (higher gonadotropin levels) were associated with lower TBS values. In multiple regression analysis, age and gonadotropin concentration were independent factors influencing TBS values in patients with primary adrenal insufficiency. Previously it has been shown, that a linear decline of 14.5% in TBS was seen between 45 and 85 years of age (6% before 65 years and 8.5% after age 65 years) (39).

The deficiency of adrenal androgens has also been suggested as an osteoporotic risk factor in patients with primary adrenal insufficiency. Gurnell et al. showed, that 12 months of DHEA-S substitution therapy slightly but significantly increased FN BMD, but not at other skeletal sites (30). In our group, DHEA-S concentration correlated significantly with BMD in all examined locations (LS, FN, total body). In accordance with the work of Gurnell et al., the associations between androgens and densitometry parameters were more visible at the FN than in other locations. Apart from DHEA-S, we found associations between FAI and FN T score, Z score and BMD, and also between testosterone and FN BMD.

To our best knowledge, this is the first study to examine sclerostin concentrations in patients with autoimmune primary adrenal insufficiency. We have found that in patients with Addison`s disease the serum sclerostin concentration was significantly higher than in the control group (p=0.006). Studies in mice and *in vitro* are rather consistent, showing increased expression of sclerostin after glucocorticoid therapy and a protective role of anti-sclerostin antibodies against glucocorticoid-induced osteoporosis (19, 20, 22, 40), but in humans, results are so far contradictory. Gifre et al. showed an increase in sclerostin concentrations in patients treated for a year with prednisone (mostly due to hematological diseases). Moreover, sclerostin was correlated with glucocorticoid dose (23). Other studies comprising healthy patients treated with prednisone for a shorter period (25) or patients with endogenous hypercortisolemia (24) reported a glucocorticoid-related decrease in sclerostin concentrations. In this context, our result is very interesting. Our study group was homogenous, treated with hydrocortisone for a long time, and presented no decrease in BMD or TBS in comparison to controls, so the difference in sclerostin concentrations was probably not an effect of the changes in osteocyte number, which are the main source of sclerostin. There was a significant correlation between serum sclerostin and fT4 concentration in patients with primary adrenal insufficiency. There was no correlation with any other parameters (including TSH, daily dose of hydrocortisone or thyroxine, UFC), so the relevance of this correlation remains unclear. Previously it has been shown that after the successful treatment of thyrotoxicosis, the level of serum sclerostin decreases (41). Also, in the group of patients with different thyroid status, serum sclerostin correlated positively with fT4 and negatively with TSH (42). In the current study, fT4 concentration did not differ between patients with Addison`s disease and controls, so we cannot draw conclusions, that different thyroid status explains higher serum sclerostin in patients with primary adrenal insufficiency. However, thyroid status is known to affect bone metabolism and most probably affects sclerostin level, this fact needs to be taken into consideration in further studies on this topic.

Since this is the first study concerning sclerostin in Addison’s disease, it is difficult to draw conclusions why sclerostin was higher in patients than in controls. We present few hypotheses of this result. Firstly, treatment with steroids may increase sclerostin concentration like in animals and some human studies. This association may occur before the impairment of bone status and may be differently pronounced depending on steroid type and duration of treatment. Maybe our study was too small to detect influence of mild hydrocortisone overtreatment on sclerostin. Also factors influencing steroid metabolism may be important, as discussed above. Secondly, the autoimmune process may promote other sources of sclerostin production. It has been suggested that in rheumatoid arthritis fibroblast-like synoviocytes were a major source of sclerostin (43). Interestingly, the autoimmune disease was present in all our patients and most of patients from the study of Gifre et al., both groups were treated with steroids for longer period, resulting in significant sclerostin increase. Also, thyroid status may affect sclerostin concentration, as discussed above. In our study group more patients than controls were treated with L-thyroxine and sclerostin correlated with fT4 concentration. However, fT4 did not differ significantly between patients and controls, so this aspect needs further studies.

In this context, we believe that our study should be considered as preliminary one, we are planning follow up. The strength of our work is the homogeneity of our study group (autoimmune etiology of the disease, treatment with hydrocortisone) and the use of novel methods (first report of TBS and sclerostin in Addison`s disease). The limitations of our study are low number of patients and lack of radiological imaging focused on osteoporotic fractures. This topic needs further studies, especially multicenter, with greater number of patients, with follow up and radiological assessment of fractures. To further explore the reasons of sclerostin alterations, studies could assess also patients without autoimmune disease (for example after bilateral adrenalectomy).

In conclusion, we performed the first published study comparing TBS values and sclerostin concentrations between patients with autoimmune primary adrenal insufficiency and healthy controls. TBS results were not impaired in patients with Addison`s disease while sclerostin concentrations were significantly increased in comparison to healthy subjects.

## Data availability statement

The raw data supporting the conclusions of this article will be made available by the authors, without undue reservation.

## Ethics statement

The studies involving human participants were reviewed and approved by The Bioethics Committee of Wroclaw Medical University. The patients/participants provided their written informed consent to participate in this study.

## Author contributions

All authors contributed to the study conception and design. Material preparation, data collection, and analysis were performed by AZ-W, JS, and JH-Z. The first draft of the manuscript was written by AZ-W, and all authors commented on previous versions of the manuscript. AZ-W conceptualized the study. JH-Z contributed to the methodology. JS performed TBS examination and analysis. NS performed the sclerostin examination and analysis. AZ-W, JH-Z, ŁG, and MB performed the Formal analysis and investigation. AZ-W and JH-Z wrote and prepared the original draft. ŁG, JS, and MB wrote, reviewed, and edited the manuscript. All authors contributed to the article and approved the submitted version.

## Funding

This work was supported by Wroclaw Medical University grant for young researchers, number: STM.C120.20.042.

## Conflict of interest

The authors declare that the research was conducted in the absence of any commercial or financial relationships that could be construed as a potential conflict of interest.

## Publisher’s note

All claims expressed in this article are solely those of the authors and do not necessarily represent those of their affiliated organizations, or those of the publisher, the editors and the reviewers. Any product that may be evaluated in this article, or claim that may be made by its manufacturer, is not guaranteed or endorsed by the publisher.

## References

[1] Laureti S Vecchi L Santeusanio F Falorni A. Is the prevalence of addison’s disease underestimated? J Clin Endocrinol Metab (1999) 84(5):1762–2. doi: 10.1210/jcem.84.5.5677-7 10323417

[2] Løvås K Husebye ES. High prevalence and increasing incidence of addison’s disease in western Norway. Clin Endocrinol (Oxf). (2002) 56(6):787–91. doi: 10.1046/j.1365-2265.2002.t01-1-01552.x 12072049

[3] Meyer G Neumann K Badenhoop K Linder R. Increasing prevalence of addison’s disease in German females: health insurance data 2008–2012. Eur J Endocrinol (2014) 170(3):367–73. doi: 10.1530/EJE-13-0756 24322183

[4] Løvås K Loge JH Husebye ES. Subjective health status in Norwegian patients with addison’s disease. Clin Endocrinol (Oxf). (2002) 56(5):581–8. doi: 10.1046/j.1365-2265.2002.01466.x 12030907

[5] Meyer G Hackemann A Penna-Martinez M Badenhoop K. What affects the quality of life in autoimmune addison’s disease? Horm Metab Res Horm Stoffwechselforschung Horm Metab (2013) 45(2):92–5. doi: 10.1055/s-0032-1331766 23322510

[6] Hahner S Loeffler M Fassnacht M Weismann D Koschker AC Quinkler M. Impaired subjective health status in 256 patients with adrenal insufficiency on standard therapy based on cross-sectional analysis. J Clin Endocrinol Metab (2007) 92(10):3912–22. doi: 10.1210/jc.2007-0685 17684047

[7] Bergthorsdottir R Leonsson-Zachrisson M Odén A Johannsson G. Premature mortality in patients with addison’s disease: a population-based study. J Clin Endocrinol Metab (2006) 91(12):4849–53. doi: 10.1210/jc.2006-0076 16968806

[8] Erichsen MM Løvås K Fougner KJ Svartberg J Hauge ER Bollerslev J. Normal overall mortality rate in addison’s disease, but young patients are at risk of premature death. Eur J Endocrinol (2009) 160(2):233–7. doi: 10.1530/EJE-08-0550 19011006

[9] Bensing S Brandt L Tabaroj F Sjöberg O Nilsson B Ekbom A. Increased death risk and altered cancer incidence pattern in patients with isolated or combined autoimmune primary adrenocortical insufficiency. Clin Endocrinol (Oxf). (2008) 69(5):697–704. doi: 10.1111/j.1365-2265.2008.03340.x 18727712

[10] Dalin F Nordling Eriksson G Dahlqvist P Hallgren Å Wahlberg J Ekwall O. Clinical and immunological characteristics of autoimmune Addison disease: A nationwide Swedish multicenter study. J Clin Endocrinol Metab (2017) 102(2):379–89. doi: 10.1210/jc.2016-2522 27870550

[11] Bergthorsdottir R Ragnarsson O Skrtic S Glad CAM Nilsson S Ross IL. Visceral fat and novel biomarkers of cardiovascular disease in patients with addison’s disease: A case-control study. J Clin Endocrinol Metab (2017) 102(11):4264–72. doi: 10.1210/jc.2017-01324 28945861

[12] Løvås K Gjesdal CG Christensen M Wolff AB Almås B Svartberg J. Glucocorticoid replacement therapy and pharmacogenetics in addison’s disease: effects on bone. Eur J Endocrinol (2009) 160(6):993–1002. doi: 10.1530/EJE-08-0880 19282465

[13] Björnsdottir S Sääf M Bensing S Kämpe O Michaëlsson K Ludvigsson JF. Risk of hip fracture in addison’s disease: a population-based cohort study. J Intern Med (2011) 270(2):187–95. doi: 10.1111/j.1365-2796.2011.02352.x 21251095

[14] Arlt W Rosenthal C Hahner S Allolio B. Quality of glucocorticoid replacement in adrenal insufficiency: clinical assessment vs. timed serum cortisol measurements. Clin Endocrinol (Oxf). (2006) 64(4):384–9. doi: 10.1111/j.1365-2265.2006.02473.x 16584509

[15] Jódar E Valdepeñas MPR Martinez G Jara A Hawkins F. Long-term follow-up of bone mineral density in addison’s disease. Clin Endocrinol (Oxf). (2003) 58(5):617–20. doi: 10.1046/j.1365-2265.2003.01761.x 12699444

[16] Esteban NV Loughlin T Yergey AL Zawadzki JK Booth JD Winterer JC. Daily cortisol production rate in man determined by stable isotope dilution/mass spectrometry. J Clin Endocrinol Metab (1991) 72(1):39–45. doi: 10.1210/jcem-72-1-39 1986026

[17] Hardy RS Zhou H Seibel MJ Cooper MS. Glucocorticoids and bone: Consequences of endogenous and exogenous excess and replacement therapy. Endocr Rev (2018) 39(5):519–48. doi: 10.1210/er.2018-00097 29905835

[18] Delgado-Calle J Sato AY Bellido T. Role and mechanism of action of sclerostin in bone. Bone (2017) 96:29–37. doi: 10.1016/j.bone.2016.10.007 27742498 PMC5328835

[19] Morimoto E Li M Khalid AB Krum SA Chimge NO Frenkel B. Glucocorticoids hijack Runx2 to stimulate Wif1 for suppression of osteoblast growth and differentiation. J Cell Physiol (2017) 232(1):145–53. doi: 10.1002/jcp.25399 PMC5540653 27061521

[20] Ohnaka K Tanabe M Kawate H Nawata H Takayanagi R. Glucocorticoid suppresses the canonical wnt signal in cultured human osteoblasts. Biochem Biophys Res Commun (2005) 329(1):177–81. doi: 10.1016/j.bbrc.2005.01.117 15721290

[21] Sato AY Cregor M Delgado-Calle J Condon KW Allen MR Peacock M. Protection from glucocorticoid-induced osteoporosis by anti-catabolic signaling in the absence of Sost/Sclerostin. J Bone Miner Res Off J Am Soc Bone Miner Res (2016) 31(10):1791–802. doi: 10.1002/jbmr.2869 PMC8499032 27163932

[22] Yao W Dai W Jiang L Lay EYA Zhong Z Ritchie RO. Sclerostin-antibody treatment of glucocorticoid-induced osteoporosis maintained bone mass and strength. Osteoporos Int J Establ Result Coop Eur Found Osteoporos Natl Osteoporos Found USA (2016) 27(1):283–94. doi: 10.1007/s00198-015-3308-6 PMC4958115 26384674

[23] Gifre L Ruiz-Gaspà S Monegal A Nomdedeu B Filella X Guañabens N. Effect of glucocorticoid treatment on wnt signalling antagonists (sclerostin and dkk-1) and their relationship with bone turnover. Bone (2013) 57(1):272–6. doi: 10.1016/j.bone.2013.08.016 23981659

[24] van Lierop AH van der Eerden AW Hamdy N a. T Hermus AR den Heijer M Papapoulos SE. Circulating sclerostin levels are decreased in patients with endogenous hypercortisolism and increase after treatment. J Clin Endocrinol Metab (2012) 97(10):E1953–1957. doi: 10.1210/jc.2012-2218 PMC3462943 22844062

[25] Jacobsson M Raalte DH Heijboer AC Heijer M Jongh RT. Short-term glucocorticoid treatment reduces circulating sclerostin concentrations in healthy young men: A randomized, placebo-controlled, double-blind study. JBMR Plus (2020) 4(8)1–6. doi: 10.1002/jbm4.10341 PMC7422706 32803106

[26] Zaheer S Meyer K Easly R Bayomy O Leung J Koefoed AW. Effect of adrenocorticotropic hormone infusion on circulating sclerostin levels. Endocr Connect. (2021) 10(12):1607–14. doi: 10.1530/EC-21-0263 PMC8679878 34788228

[27] Shin YH Gong HS Lee KJ Baek GH. Older age and higher body mass index are associated with a more degraded trabecular bone score compared to bone mineral density. J Clin Densitom. (2019) 22(2):266–71. doi: 10.1016/j.jocd.2017.06.006 28712983

[28] Saag KG Agnusdei D Hans D Kohlmeier LA Krohn KD Leib ES. Trabecular bone score in patients with chronic glucocorticoid therapy-induced osteoporosis treated with alendronate or teriparatide: Trabecular bone score in glucocorticoid-induced osteoporosis. Arthritis Rheumatol (2016) 68(9):2122–8. doi: 10.1002/art.39726 27111239

[29] Stachowska B Halupczok-Żyła J Kuliczkowska-Płaksej J Syrycka J Bolanowski M. Decreased trabecular bone score in patients with active endogenous cushing’s syndrome. Front Endocrinol (2020) 11:593173. doi: 10.3389/fendo.2020.593173 PMC7874075 33584537

[30] Gurnell EM Hunt PJ Curran SE Conway CL Pullenayegum EM Huppert FA. Long-term DHEA replacement in primary adrenal insufficiency: a randomized, controlled trial. J Clin Endocrinol Metab (2008) 93(2):400–9. doi: 10.1210/jc.2007-1134 PMC2729149 18000094

[31] Bornstein SR Allolio B Arlt W Barthel A Don-Wauchope A Hammer GD. Diagnosis and treatment of primary adrenal insufficiency: An endocrine society clinical practice guideline. J Clin Endocrinol Metab (2016) 101(2):364–89. doi: 10.1210/jc.2015-1710 PMC4880116 26760044

[32] Dimai HP. Use of dual-energy X-ray absorptiometry (DXA) for diagnosis and fracture risk assessment; WHO-criteria, T- and z-score, and reference databases. Bone (2017) 104:39–43. doi: 10.1016/j.bone.2016.12.016 28041872

[33] Dufour R Winzenrieth R Heraud A Hans D Mehsen N. Generation and validation of a normative, age-specific reference curve for lumbar spine trabecular bone score (TBS) in French women. Osteoporos Int (2013) 24(11):2837–46. doi: 10.1007/s00198-013-2384-8 23681084

[34] Camozzi V Betterle C Frigo AC Zaccariotto V Zaninotto M De Caneva E. Vertebral fractures assessed with dual-energy X-ray absorptiometry in patients with addison’s disease on glucocorticoid and mineralocorticoid replacement therapy. Endocrine (2018) 59(2):319–29. doi: 10.1007/s12020-017-1380-8 28795340

[35] Crown A Lightman S. Why is the management of glucocorticoid deficiency still controversial: a review of the literature. Clin Endocrinol (Oxf). (2005) 63(5):483–92. doi: 10.1111/j.1365-2265.2005.02320.x 16268798

[36] Murray RD Ekman B Uddin S Marelli C Quinkler M Zelissen PMJ. Management of glucocorticoid replacement in adrenal insufficiency shows notable heterogeneity - data from the EU-AIR. Clin Endocrinol (Oxf). (2017) 86(3):340–6. doi: 10.1111/cen.13267 27801983

[37] Espiard S McQueen J Sherlock M Ragnarsson O Bergthorsdottir R Burman P. Improved urinary cortisol metabolome in Addison disease: A prospective trial of dual-release hydrocortisone. J Clin Endocrinol Metab (2021) 106(3):814–25. doi: 10.1210/clinem/dgaa862 PMC7947853 33236103

[38] Shevroja E Lamy O Kohlmeier L Koromani F Rivadeneira F Hans D. Use of trabecular bone score (TBS) as a complementary approach to dual-energy X-ray absorptiometry (DXA) for fracture risk assessment in clinical practice. J Clin Densitom. (2017) 20(3):334–45. doi: 10.1016/j.jocd.2017.06.019 28734710

[39] Silva BC Leslie WD Resch H Lamy O Lesnyak O Binkley N. Trabecular bone score: A noninvasive analytical method based upon the DXA image: TRABECULAR BONE SCORE. J Bone Miner Res (2014) 29(3):518–30. doi: 10.1002/jbmr.2176 24443324

[40] Beier EE Sheu TJ Resseguie EA Takahata M Awad HA Cory-Slechta DA. Sclerostin activity plays a key role in the negative effect of glucocorticoid signaling on osteoblast function in mice. Bone Res (2017) 5(1)1–14. doi: 10.1038/boneres.2017.13 PMC5422922 28529816

[41] Skowrońska-Jóźwiak E Krawczyk-Rusiecka K Lewandowski KC Adamczewski Z Lewiński A. Successful treatment of thyrotoxicosis is accompanied by a decrease in serum sclerostin levels. Thyroid Res (2012) 5(1)1–3. doi: 10.1186/1756-6614-5-14 PMC3537580 23146624

[42] Mihaljević O Živančević-Simonović S Lučić-Tomić A Živković I Minić R Mijatović-Teodorović L. The association of circulating sclerostin level with markers of bone metabolism in patients with thyroid dysfunction. J Med Biochem (2020) 39(4):436–43. doi: 10.5937/jomb0-24943 PMC7710371 33312059

[43] Wehmeyer C Frank S Beckmann D Böttcher M Cromme C König U. Sclerostin inhibition promotes TNF-dependent inflammatory joint destruction. Sci Transl Med (2016) 8(330):330ra35. doi: 10.1126/scitranslmed.aac4351 27089204

